# Comparative analysis of extraction technologies for plant extracts and absolutes

**DOI:** 10.3389/fchem.2025.1536590

**Published:** 2025-03-03

**Authors:** Shoutao Cao, Jinchang Liang, Mingguang Chen, Chao Xu, Xiaoqiang Wang, Lei Qiu, Xianyan Zhao, Wenxiao Hu

**Affiliations:** ^1^ State Key Laboratory of Bio-Based Materials and Green Paper Making, Qilu University of Technology (Shandong Academy of Sciences), Jinan, China; ^2^ Department of Plant Protection, Tobacco Research Institute of Chinese Academy of Agricultural Sciences, Qingdao, China

**Keywords:** plant extract, plant absolute, conventional extraction technology, green extraction techniques, green extraction solvent

## Abstract

Plant extracts and absolutes have high application value in several industries such as medicine, food, and fragrance. Especially in the field of fragrance, while there is expensive, they are prized by perfumers and provide a rich and lasting aroma. Owing to advancements in extraction technology, their yields have increased and their ingredients have become richer. However, no extraction technology is universal and each extraction technology has its own distinct advantages and disadvantages. Therefore, this review systematically characterizes the extraction technologies for plant extracts and absolutes, including traditional extraction technologies, such as maceration, percolation, reflux, and Soxhlet extraction, and green extraction technologies, such as microwave-assisted, ultrasonic-assisted, pressurized liquid, and supercritical fluid extractions. These extraction technologies are analyzed and compared in terms of their principles, advantages and disadvantages, improvement solutions, and applications. In addition, this review summarizes and compares new green extraction solvents and discusses the practical applications of these advanced extraction methods and solvents from different perspectives.

## 1 Introduction

Plant extraction involves the use of solvents to extract various plant organs and tissues, such as flowers, leaves, and fruits. The extracted mixture is then subjected to filtration, evaporation, and other processes to obtain a thick paste-like liquid with a distinct aroma and abundant biologically active compounds, which is defined as plant extracts (Zhao, 2022; Azmir et al., 2013; Aftab and Hakeem). This liquid serves as an essential precursor for producing plant absolutes, which are clear liquids obtained via the extraction of plant extracts, balsams, or supercritical fluids using high-purity ethanol (Fernandez et al., 2005). Plant extracts and absolutes are commonly used as natural plant fragrances and primarily comprise olefins, alcohols, aldehydes, ketones, and other aromatic compounds (Zhou et al., 2022; Derbassi et al., 2022; Balci-Torun and Ozdemir, 2021). Plant extracts or absolutes may not have the same scent as the original plant; however, they tend to have a more intricate and superior aroma (Rout et al., 2011). Thus, these high-quality flavoring agents are commonly used in the fragrance industry. In addition, plant extracts and absolutes contain various biologically active ingredients and are widely used in the pharmaceutical industry.

During the preparation of plant extracts and absolutes, the quality and speed of extraction are significantly affected by the extraction process (Yang, 2021b). Currently, the primary extraction methods used in the plant extraction industry are limited to traditional organic solvent extraction methods. However, the limitations of organic solvent extraction, such as long extraction times, high energy consumption, and high toxicity, have attracted attention (Chen et al., 2018). Recently, research on environmentally friendly alternative solvents and new extraction technologies has made significant progress (Chemat et al., 2020). Various green extraction technologies, such as supercritical fluid extraction (SFE) and microwave-assisted extraction (MAE), are relatively mature and gradually being used in industrial production processes (Lefebvre et al., 2021). However, each extraction technology has its own characteristics, advantages, and limitations. Choosing the right extraction process is the key to improving the yield and quality of plant extracts and absolutes (Joana Gill-Chavez et al., 2013; Tiwari, 2015). Numerous studies indicate that using a combination of new extraction strategies leads to faster and more efficient extraction (Chemat et al., 2017). From this perspective, this review comparatively analyzes the principles and characteristics of different extraction technologies and discusses possible problems and solutions in their practical applications. We hope that this will provide useful information for plant extracts and absolutes, a direction for the development of extraction technology, and references for the practical applications of green extraction technology.

## 2 Conventional extraction techniques

### 2.1 Maceration

Maceration refers to the use of low-boiling, volatile organic solvents, such as petroleum ether and tetrachloromethane, to promote a more selective and efficient mass transfer of compounds in plants via the use of various solvents, temperatures, and stirring combinations to obtain the target extract (Mohana Roopan and Madhumitha, 2018). The organic solvent is then recovered using vacuum distillation and other methods to obtain a pasty extract used to prepare the absolute (Wang et al., 2023). This method has the advantages of simple equipment and operation, a high extraction rate, and the ability to select solvents according to the target components (Garcia-Vaquero et al., 2020). For example, benzene has a high selectivity for non-polar lipid components, ethanol can extract both polar and non-polar substances, and hexane can selectively extract non-polar substances (Escorsim et al., 2018; Mercer and Armenta, 2011). These advantages have been widely exploited in the production of vegetable absolutes. However, organic solvent extraction methods are often time-consuming, particularly when using large volumes of toxic organic solvents, which also increases the potential safety hazards for production workers and food consumers exposed to these chemicals (Garcia-Vaquero et al., 2020). Although researchers have reported the safety issues of solvent extraction, current research and the utilization of this method continue owing to its simplicity and high extraction rate. Tian et al. (2023) used organic solvents (petroleum ether, absolute ethanol, butanol, and ethyl acetate) to extract violet extract. Subsequently, they used high-pressure extraction to obtain violet absolute, which was used as a fragrance base module to produce cigarette explosion beads. Yang (2021a) used petroleum ether as a solvent to extract Osmanthus extract and obtained Osmanthus absolute using vacuum fractionation. Following aroma identification, the aroma was found to be more similar to that of natural Osmanthus, and the fragrance lasted longer.

### 2.2 Percolation

**FIGURE 1:**
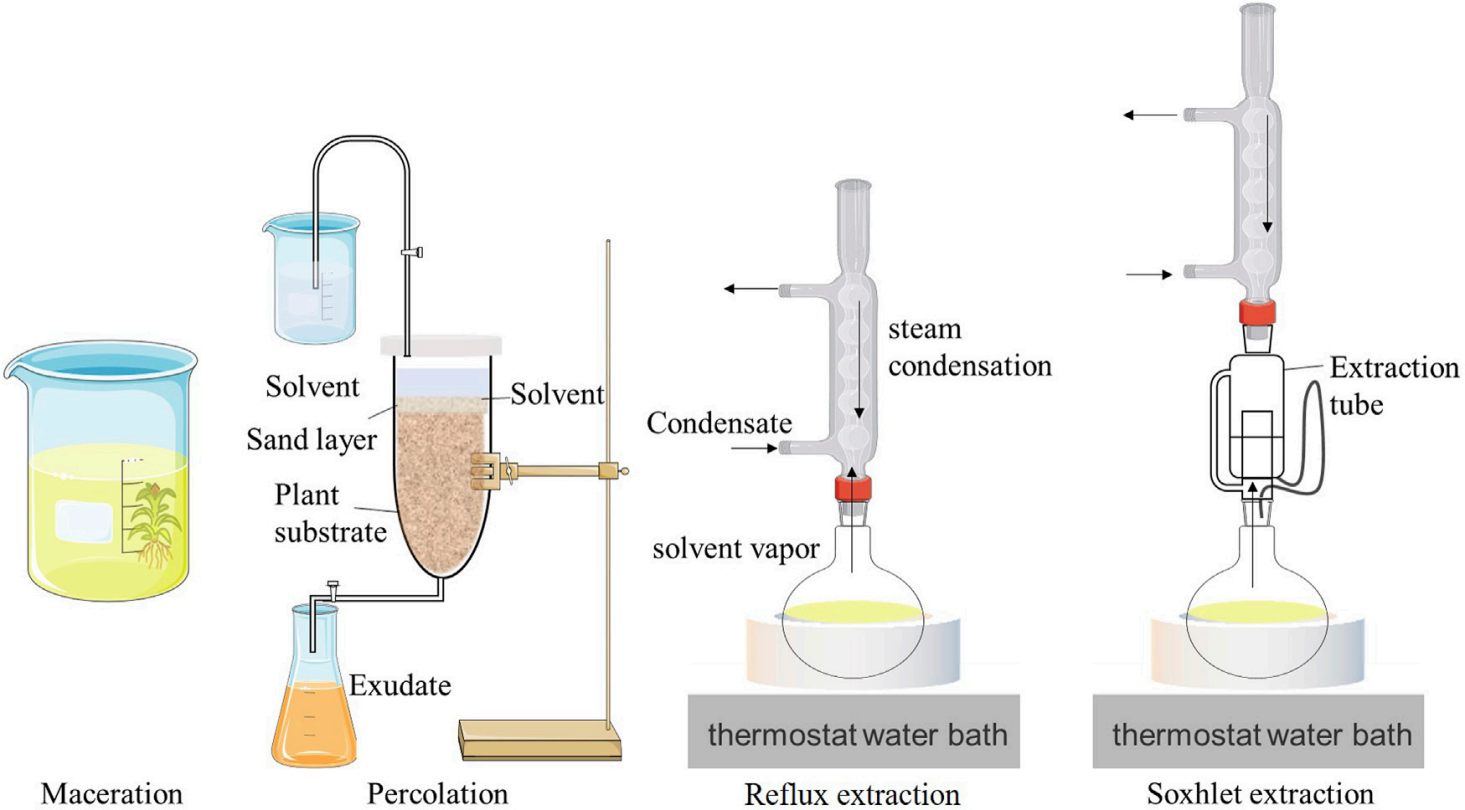
Schematic diagrams of the extraction equipment for four conventional extraction techniques.

As shown in Figure 1, percolation uses a dynamic leaching technology based on maceration that continuously adds new solvents to maintain a certain concentration difference during the extraction process, thereby improving the extraction efficiency. However, it further increases solvent consumption. Percolation is generally suitable for valuable and toxic compounds and high-concentration preparations (Wang et al., 2020). Examples of percolation include the production of traditional Chinese medicine extracts, such as belladonna (Zhang et al., 2022) and *Polygala extracts* (Xu et al., 2020), and the extraction of cigarette extracts, such as perilla seed (Wu and Huang, 2023) and chrysanthemum flower extracts (Nong and Wu, 2020).

### 2.3 Reflux extraction

Reflux extraction uses a reflux device, as shown in Figure 1, to repeatedly heat and reflux the volatile extraction solvent until the active ingredients in the raw material are completely extracted. While improving extraction efficiency, it avoids the volatilization loss of a large volume of solvent and the discharge of toxic solvents (Cai and Xu, 2023). However, this extraction method has a low extraction efficiency for numerous non-volatile active ingredients that are present in plant extracts or absolutes. In addition, some thermally unstable components are destroyed during the heating process. Therefore, the reflux extraction method is often used to extract volatile components, such as flavonoids (Han and Jiao, 2022) and saponins (Cai and Zhang, 2023), from natural plants.

### 2.4 Soxhlet extraction

Soxhlet extraction, also known as continuous extraction, is a classic method for extracting compounds from solid substances. As shown in Figure 1, Soxhlet extraction uses the principles of solvent reflux and siphoning to enable the extraction of solid substances using pure solvents, thereby improving extraction efficiency. The solvent in the extraction chamber rises along the air conduit following heating and boiling, condenses into liquid, and drips into the extractor. When the liquid level exceeds the highest point in the siphon tube, the solution is drawn into the flask. This process is re-peated until complete extraction occurs (Luque de Castro and Priego-Capote, 2010). Compared to the leaching method, the Soxhlet extraction method has certain advantages. First, the fresh solvent continues to flow to the sample, which is beneficial for improving mass transfer during compound extraction. Second, it ensures a thermal effect on the sample. Finally, it can achieve a relatively low cost and ease of operation for multiple samples (Wang and Weller, 2006). Owing to these advantages, the Soxhlet extraction method has also been applied to plant extracts. For example, Hu and Hu (2021) optimized the process of extracting *S. grosvenorii* extract using Soxhlet extraction. The total content of key aroma components of *Siraitia grosvenorii* attained 12.81 mg/g. Gao and Wang. (2019) used the Soxhlet extraction method to extract mulberry leaf extract with an extraction rate of 1.80%, and the extract showed good antioxidant and antibacterial activities. However, limitations such as long extraction times, the easy degradation of high-value compounds during the extraction process, and the use of toxic organic solvents restrict its development and application.

In summary, traditional extraction techniques have limitations such as the use of toxic solvents and large dosages, long extraction times, multiple exhaust processes, and low extraction yields. Particularly in the tobacco flavoring industry, although the extract and absolute oil extracted using traditional extraction methods can better compensate for tobacco aroma, the residue of the solvent is accompanied by discomfort and roughness during smoking and negative effects such as excessive tongue astringency (Tian and Zhao, 2023). Industrial demands for high quality, high output, low cost, low energy consumption, and environmental friendliness have brought new challenges to the research and development of extraction technologies for plant extracts and absolutes.

## 3 Green extraction techniques

Compared to traditional extraction technology, green extraction technology can use green extraction solvents to replace toxic extraction solvents, thereby avoiding the presence of toxic components in products and the environment. Moreover, it can improve the extraction rate using auxiliary extraction technologies (microwaves, ultrasonics, and pressure), shorten the extraction time, and reduce solvent and energy consumption.

### 3.1 Green alternative solvents

For certain natural plants, organic solvent extraction is more conducive for the large-scale production of plant extracts and absolutes. For example, the low cost and high solubility of n-hexane render it widely used on an industrial scale. However, it is nonrenewable and significantly impacts the environment, which are limitations that are difficult to overcome. In addition, traditional organic solvents may have certain chemical smells, such as an ether smell, which not only affect the smell of the plant extract and absolute, but are also toxic (Drozi and David, 2023). Therefore, several alternative green solvents have been developed and gradually applied in extraction processes. Based on the Twelve Principles of Green Chemistry, green solvents must meet the following criteria: availability (low fluctuations in production capacity), competitive price, recyclability, high quality, easily obtained, non-toxic, biodegradable, highly efficient, thermally and chemically stable, non-flammable, easily stored, and renewable (Şahin, 2019).

Ionic liquids (ILs) were the first batch of green alternative solvents developed and comprise a mixture of two ionic compounds with opposite charges that produce different physical and chemical properties from the compounds used. The ability of ILs to improve the extraction rate has also been demonstrated. The addition of salt ions strengthens the ion exchange process in the solution, rendering it easier for certain plant components (such as alkaloids) to spread into the solvent. Moreover, ILs can be used to extract compounds that cannot be extracted by other selective solvents (Lefebvre et al., 2021; Passos et al., 2014). The detailed extraction mechanism can be explained in two steps. First, the IL complexes with the cellulose in the plant cell wall, breaking the hydrogen bonds between the cellulose molecules and making the cellulose soluble in the IL. Second, the designed IL interacts with groups of the target extract *via* electrostatic, dispersion, and hydro-gen bonding, which further increases the solubility of the target extract (Guo and Han, 2023). Therefore, ILs are more commonly used to extract specific compounds. However, minimal studies have reported on multi-component extracts, such as plant extracts or absolutes. For example, He and He (2023) extracted tobacco extract using a 1-octyl-3-methyl-imidazolium bromide ion solution, and Li and Ren (2023) used choline hydroxide and arginine to extract *Centella asiatica* extract. Based on the mechanism of destroying plant cellulose, using ILs is a promising approach to extract plant extracts and absolutes. In particular, for tobacco flavors and fragrances, the extraction of key aromatic components can be further enhanced through targeted design.

However, the preparation costs of ionic liquids are considerably higher than those of traditional organic solvents, which makes large-scale applications difficult. Furthermore, their toxicity can sometimes be greater than that of organic solvents, and they are more resistant to biodegradation. As a result, the “green” properties of ionic liquids are still debatable (Thuy Pham et al., 2010; Cvjetko Bubalo et al., 2014).

Natural deep eutectic solvents (NaDESs) are green substitutes for traditional solvents and are a type of deep eutectic solvent (DES). They primarily refer to the solvent synthesized using primary metabolites, such as organic acids, sugars, amino acids, or choline derivatives, as hydrogen bond donors and receptors, which are liquids at room temperature. With the characteristics of low toxicity, recyclability, low cost, environmentally friendliness, and adjustable properties, it is termed “the new green solvent in the 21st century” (Bosiljkov et al., 2017). Typical representatives of NaDESs are non-toxic choline chloride and naturally uncharged compounds such as alcohols, amines, vitamins, carboxylic acids, and sugars, which can be used as hydrogen bond donors. By changing the composition of the DES hydrogen bond donors and acceptors, the solubility, melting point, density, viscosity, electrical conductivity, and toxicity of the solvents can be adjusted, which is convenient for the selection and preparation of suitable solvents (Paiva et al., 2014; Xuan and Zhuang, 2021). Lanjekar and Rathod (2021) combined supercritical CO_2_ fluid (scCO_2_) with NaDESs to extract aroma components of lavender. The effects of the four NaDESs on the scCO_2_ extract were compared and analyzed. All four types of supercritical CO_2_ could be used as stable media for scCO_2_ extraction and could ensure the stability of the aroma components of the lavender extract for an extended period. Su and Chen (2022) used butanediol and levulinic acid at a molar ratio of 2:1 to prepare a DES for the extraction of *Ginkgo biloba* extract. Under the action of DESs, the extraction rate of various components (such as flavonoids, terpenes, and esters) increased by more than 100 times. However, DESs often have high viscosities and densities, which hinder the mass transfer effect of the solvent and is a crucial problem that must be solved (Şahin, 2019).

Deep eutectic solvents represent a new generation of ionic liquids and share many of their advantages. For example, compared to traditional solvents, they have favorable thermal properties and a broad liquid range, remaining stable even at temperatures as high as 100°C. Moreover, they exhibit a wide range of solubility and demonstrate efficient recovery performance (Şahin, 2019). Natural deep eutectic solvents have addressed some of the drawbacks associated with ionic liquids, such as higher toxicity and lower biodegradability. However, they still face the challenge of high preparation costs. In recent years, advancements in ionic liquid technology have significantly improved the recovery rates of these solvents. Therefore, reducing costs through recycling and reuse is a crucial strategy for making the extraction of plant absolutes using ionic liquids economically viable (Meksi and Moussa 2017; Isosaari et al., 2019; Ventura et al., 2017). In comparison to traditional solvent extraction, ionic liquids offer shorter extraction times (Bonny et al., 2011), higher extraction efficiency (Bogdanov et al., 2015), and lower energy consumption (Fan et al., 2018), making them a promising green alternative solvent.

In recent years, many studies have explored the combination of ionic liquids with new green extraction technologies. This approach can enhance extraction efficiency, lower solvent consumption, and reduce costs. For example, when combining ionic liquids with microwave-assisted extraction technology, the hydrogen bonding interaction between the ionic liquid and the target component helps to enrich the desired substances. Additionally, ionic liquids effectively transmit microwave energy, further improving the extraction efficiency of those components (Lim et al., 2022; Tan et al., 2016). For instance, Bonny et al. demonstrated that the extraction efficiency of Norstic acid using Ionic Liquid Microwave-Assisted Extraction (IL-MAE) is 1.5–3 times higher than that of Ionic Liquid heat-reflux extraction (Bonny et al., 2011). Additionally, Zhao et al. (2019) discovered that by combining Deep Eutectic Solvents (DES) with Microwave-Assisted Extraction (MAE) for pretreatment, both the extraction rate and the variety of extracted compounds significantly improved compared to using MAE alone. Therefore, combining green alternative solvents with various green extraction technologies will be an important research direction for the development of extraction technology in the future.

Aqueous cyclodextrin (CD) solutions are a new green alternative solvent that can selectively form inclusion complexes of certain sizes and polar molecules. They have the advantages of improving the solubility and biocompatibility of functional materials and are widely used in the pharmaceutical industry. Forming an inclusion complex with the components of a medicinal plant extract can improve its solubility, thus further broadening its application. For example, Liu and Yang (2023) used CDs to form an inclusion complex with some components in the extract of Flos Lonicerae, which significantly increased the solubility of these components, thus improving the extraction rate while stabilizing the dissolved solution system. In addition, several studies have confirmed that CDs are of great significance for the preservation of aroma components in plant extracts because they can minimize the volatilization and escape while ensuring the integrity of aroma components. In theory, CDs have significant application prospects for the extraction of plant extracts and the production of pure plant oil. Moreover, they are beneficial for the long-term preservation of aroma components in extracts or pure oils. However, empirical research on the use of CDs to extract extracts or pure oils is lacking, and the effects of CDs on the extraction of different components from complex systems remain to be confirmed.

Although its application is limited because it requires specialized operating equipment (such as supercritical equipment), scCO_2_ is also used as an alternative solvent. CO_2_ is typically used as a supercritical fluid in supercritical extraction because it is inert, nontoxic, and can be extracted at low temperatures and pressures. CO_2_ is a non-polar and esterophilic solvent; however, it is a weak solvent for highly polar compounds (such as phenolic compounds). However, Reetz et al. (2003) proposed the combination of ILs and scCO_2_ fluid to form a two-phase separation system as a solution. During the IL phase reaction, the target compound was extracted to the supercritical phase, and the IL was almost insoluble in scCO_2_, thereby avoiding solvent loss. In addition, ILs can increase the selectivity of scCO_2_ by certain components (Reetz et al., 2003; Hiraga et al., 2018). Although this scheme is theoretically feasible, ILs are difficult to use in practical applications because of their high cost and biodegradability. Compared with ILs, NaDESs have similar properties and are inexpensive, and their polarity is easily regulated by CO_2_ molecules, which can improve the extraction rate of more components, rendering them a better alternative (Vahidi et al., 2023).

In addition, several renewable solvents can be used as green solvents, which include water, alcohols, and ketones, that can be recycled using simple physical methods. For a more intuitive comparative analysis of the differences between the different green extraction solvents, their advantages and disadvantages are presented in Table 1.

**TABLE 1:** Comparison of different green alternative solvents.

Green alternative solvent	Advantages	Disadvantages	Remarks
IL	high extraction rate, mild processing conditions, targeted design for target components, green and environmentally friendly	complex synthesis process, high cost, produces residue, difficult recovery	IL is used as a green solvent primarily because it is non-volatile, but certain ILs (such as imidazoles) are toxic
NaDESs	high extraction rate, mild processing conditions, non-toxic, recyclable, adjustable physical and chemical properties, green and environmentally friendly	high solvent consumption, high viscosity and density	NaDESs can ensure low toxicity or even non-toxicity
CDs	high extraction rate, low cost, green and environmentally friendly	high solvent consumption, difficult to recover	CDs can not only increase the extraction rate, but also reduce the loss of volatile components
CO_2_	inert, non-toxic, mild processing conditions, easily recycled, green and environmentally friendly	requires complex equipment, high cost	CO_2_ is a non-polar and esterophilic solvent, which is not conducive to the extraction of highly polar compounds

### 3.2 Microwave-assisted extraction

**FIGURE 2:**
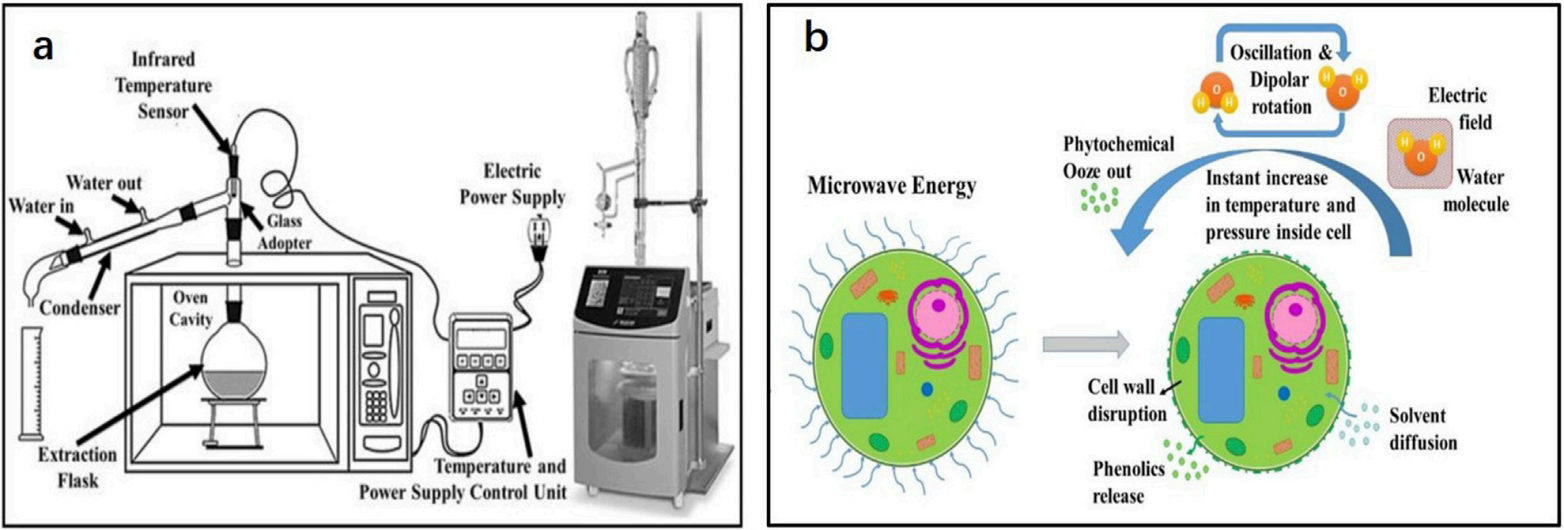
**(A)** Schematic diagram of microwave-assisted extraction method equipment; **(B)** Effect of microwaves on plant cells (Sharma and Dash, 2022; Chávez-González et al., 2020).

Microwave-assisted extraction (MAE) is a technique that uses microwave energy to stimulate the motion of liquid molecules. The equipment required for MAE is shown in Figure 2A. Under electromagnetic radiation, the electrophoretic transfer of ions and electrons initiates ion conduction to produce an electric field. Electric field dielectric heating causes the displacement of polar molecules, which results in a dipole rotation that forces the molecules to align with the existing electric field. The energy generated in the process is released in the form of heat (Marić et al., 2018). As shown in Figure 2B, the water in plant cells is heated and evaporated to form pressure, resulting in the expansion and rupture of plant cells, thereby exposing plant cells to the surrounding solvents and promoting solvent penetration to extract the target components more effectively (López-Salazar et al., 2023).

Compared with traditional extraction technologies, MAE has the advantages of uniform heating, short extraction time, less solvent loss, and a high degree of automation. Choosing an appropriate solvent based on the extraction target is particularly important in MAE. Polar solvents tend to perform better in MAE; however, some extracts show better results in mixed organic solvents and aqueous solutions (Belwal et al., 2020), and some extracts need to be extracted without using a solvent. In addition, the solid-to-liquid ratio, which is helpful for the absorption of microwave radiation, microwave power to prevent the degradation of thermosensitive compounds while promoting diffusion, and the cytological characteristics of the plant where the target extract is located also need to be considered in MAE (López-Salazar et al., 2023). Cui and Li (2023) compared and analyzed the extraction effect of chicory extract using heating reflux and MAE heating reflux methods. Following MAE, the extract yield increased by almost 3% and the aroma quality of the extract significantly improved, indicating that MAE had a beneficial effect on the extraction of plant extracts. Bao and Liu (2022) optimized the MAE process for Gyabgon grass using the response plane method. The highest extraction rate, which was almost 4% higher than that of the control group, was obtained at a microwave power, liquid-to-material ratio, and extraction time of 440 W, 8.4%, and 68 min, respectively.

MAE has certain limitations, such as the oxidation or thermal decomposition of secondary metabolites during microwave heating, poor extraction rate of non-polar or volatile target compounds (López-Salazar et al., 2023), and the solid residue remaining in the extractor that must be additionally processed via centrifugation and filtration processes (Patel et al., 2019). Recently, some new processes have been combined with the MAE process to overcome these limitations, such as nitrogen-pressurized extraction containers to prevent secondary metabolite oxidation, vacuum extraction containers to promote mass transfer, reduced thermal decomposition and oxidation reactions, and the dynamic continuous addition of fresh extraction solvents to promote mass transfer (Saha et al., 2018).

### 3.3 Ultrasonic-assisted extraction

**FIGURE 3:**
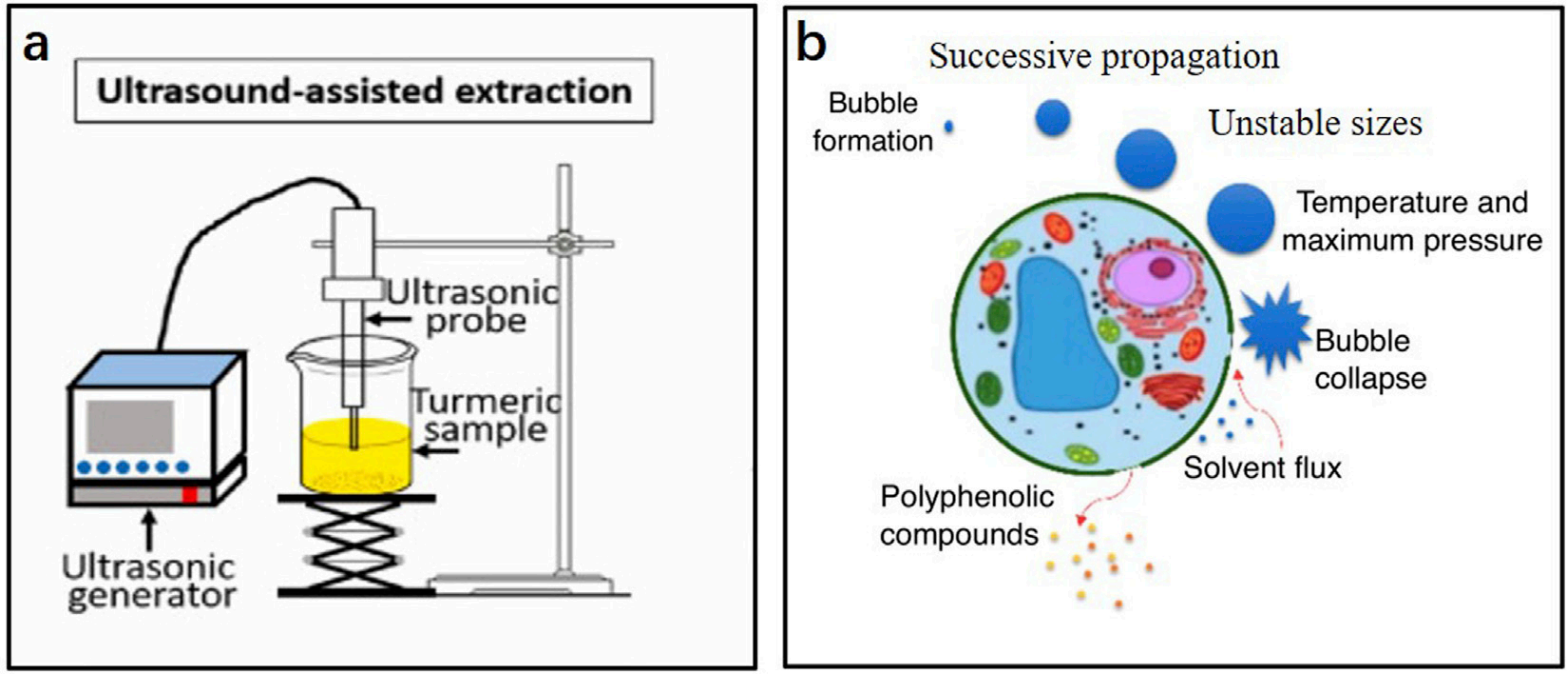
**(A)** Schematic diagram of the equipment for ultrasonic-assisted extraction; **(B)** Effect of ultrasonic waves on cells (Jiang et al., 2021; Lončarić et al., 2020).

Ultrasonic-assisted extraction (UAE) is a technology that uses an ultrasonic bath or probe to produce cavitation bubbles. The generated shear force when the cavitation bubbles burst is used to prepare the sample for extraction. A diagram of the UAE device and the effect of ultrasound on the cells are shown in Figure 3. UAE has the advantages of simple operation, low solvent consumption, short extraction time, and low process temperature. Therefore, it has potential applications in industrial plant extractions. Sun and Fei (2020) optimized the UAE process of rosemary extract using orthogonal experiments, which increased the yield of rosemary extract to 5.98%. Zhang and Pang (2016) also optimized the UAE process of olive extract using orthogonal experiments, and the extraction rate of the olive extract increased to 7.225%. Mnayer et al. (2017) utilized sunflower oil as the extraction solvent and integrated it with ultrasonic-assisted extraction technology to obtain Thymus vulgaris absolute. Their research demonstrated that this combination significantly reduced extraction time and enhanced the extraction rate, while eliminating waxy components and residues of organic solvents. Additionally, the resulting absolute oil exhibited improved antioxidant activity and a purer aroma.

However, in the UAE process, numerous reactive oxygen species are produced with the formation of cavitation bubbles, which result in the oxidation or degradation of certain compounds. Moreover, cavitation is easily affected by the physical properties of the solvent, such as its viscosity, surface tension, and saturated vapor pressure (Bachtler and Bart, 2021). Therefore, the selection of appropriate solvents is particularly important for UAE.

### 3.4 Enzyme-assisted extraction

**FIGURE 4:**
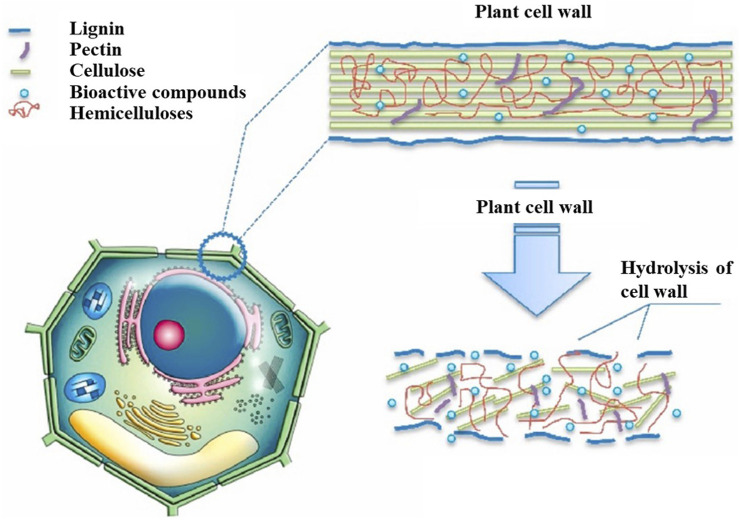
Hydrolysis of plant cell walls by enzymes (Patil et al., 2021).

Enzyme-assisted extraction (EAE) is an advanced extraction technology that increases the permeability of plant cell plasma membranes and cell walls via enzymatic hydrolysis. The enzymatic hydrolysis of the cell wall is shown in Figure 4. Several components of plant cell walls, such as lignin, cellulose, and certain proteins, not only provide cell strength but also hinder the extraction of intracellular components. Certain valuable components may exist in vacuoles or plastids or bind to the polysaccharide–lignin network via hydrogen bonding, which renders them difficult to extract with traditional methods. The commonly used enzymes in EAE are α-amylase, cellulase, pectinase, and protease. The selection of appropriate enzymes ensures that specific components are extracted from the complex substrates (Rodríguez De Luna et al., 2020; Łubek-Nguyen et al., 2022). For example, Domínguez-Rodrígueza et al. (2021) degraded pectin molecules using pectinase to extract polyphenols from sweet cherry pomace that could not be extracted using traditional methods.

EAE has the advantages of low solvent consumption, environmental friendliness, and mild processing conditions (Łubek-Nguyen et al., 2022), which not only reduce the use of harmful solvents but also increase the extraction speed and improve the extraction yield. Ling and Hu (2015) used EAE to prepare tobacco leaf extracts. After optimizing the extraction process using an orthogonal experiment, the extraction rate increased by 11.84% compared to the traditional extraction method. Bao and Piao (2022) used EAE to extract spice extract from Suijiang Kuding tea. Compared to the control group without EAE extraction, there were 19 new components. However, owing to the number of environmental requirements, high prices, and restrictions on the commercial availability of certain types of enzymes, EAE has serious limitations.

### 3.5 Supercritical fluid extraction

SFE is a technology for the extraction and separation of substances using fluids at temperatures higher than the critical temperature and pressure. Supercritical fluids have some characteristics of gas and liquid phases. For example, their densities are close to that of liquids, and their diffusion coefficient and viscosity are similar to those of gases, which endows supercritical fluids with both strong extraction ability and excellent mass transfer performance (Tian and Ma, 2023; An, 2022). Among them, scCO_2_ has become the preferred industrial extractant because of its nontoxicity, harmlessness, strong solubility, and low cost. Tian et al. (2023) used various extraction methods (such as SFE and EAE) to extract tobacco leaf extract. In their study, SFE demonstrated the shortest extraction time and a high extraction rate and avoided the tedious solvent removal process, thereby effectively retaining the original characteristic aroma of tobacco leaves. Guo and Zhang (2018) used SFE to extract rose extract, increasing the yield from 0.03% in the traditional extraction method to 0.2%, which confirmed that SFE is an effective method for extracting plant extracts.

In the process of SFE, controlling the critical temperature and optimal evaporation pressure, which increase the requirements for extraction and auxiliary equipment, are important. The simpler extraction process, lower solvent consumption, and higher extraction rate render SFE the primary method for extracting components with high commercial value. Using scCO_2_ as an example, it can only extract ester substances with low polarity and aliphatic hydrocarbons with low molecular weights, but is not ideal for hydrophilic molecules with high polarity and substances with high molecular weights (Feng and Zhang, 2022). The addition of entrainers (such as ethanol, methanol, and acetone) can further enhance the solubility and selectivity of scCO_2_ and achieve the efficient extraction of multiple target components, which is extremely important for the extraction of complex aroma components from plant extracts and absolutes. Chen and Niu (2022) studied the effects of non-entrainers and five entrainers (anhydrous ethanol, n-hexane, propylene glycol, dipropylene glycol, and dipropylene glycol methyl ether) on the SFE of tobacco extracts. Using entrainers significantly improved the extraction performance, and there were significant differences among the different entrainers. Among them, the total extraction yields of dipropylene glycol and dipropylene glycol methyl ether were high, and the yields of anhydrous ethanol and n-hexane flavor components were high. When propylene glycol was used as the entrainer, the smoke was delicate and soft. In summary, the use of an entrainer often has a beneficial effect on the extraction, and the selection of an appropriate entrainer can improve the yield of certain key components.

Another SFE method is supercritical antisolvent fractionation (SAF), which differs from SFE in that it uses scCO_2_ as the antisolvent. Because scCO_2_ does not easily dissolve polar and high-molecular-weight compounds, it can be recovered from the separation chamber and separated from non-polar components. However, SAF is often used in the treatment of extracts to further separate polar and non-polar components, and related research is scarce. In theory, it is not suitable for plant extraction, but it could be applied to the dewaxing of plant extracts for the extraction of absolutes.

### 3.6 Subcritical fluid extraction

**FIGURE 5:**
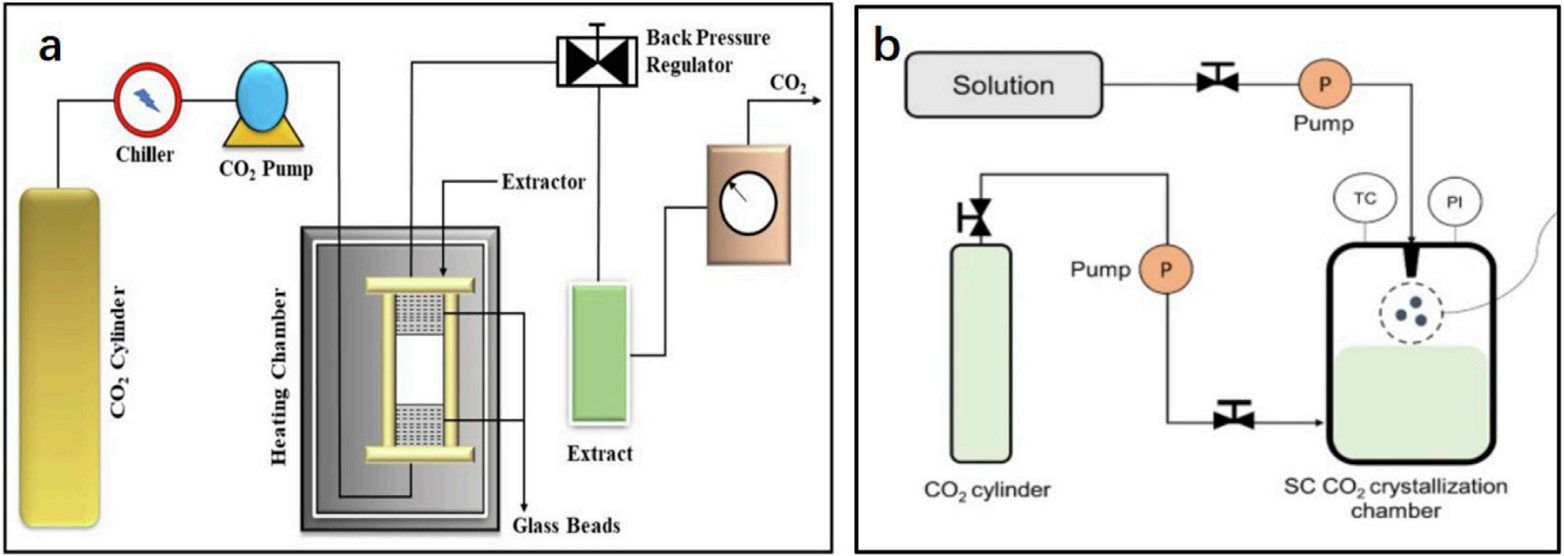
Simplified diagrams of **(A)** supercritical fluid extraction and the **(B)** supercritical antisolvent fractionation device (Kumar et al., 2022; Singh et al., 2021).

In subcritical fluid extraction (SCFE), fat-soluble components are transferred to the solvent using a subcritical solvent in an airtight, oxygen-free, low-pressure container according to a similar miscibility principle, and the extract is then obtained via vacuum evaporation (Zhu and Gan, 2020). A schematic representation of the extraction equipment is shown in Figure 5. A subcritical fluid refers to a fluid whose temperature is higher than its boiling point but lower than the critical temperature and whose pressure is lower than the critical pressure. The solvents commonly used as subcritical fluids are propane, butane, and liquid ammonia, and the boiling points are all <0°C. This ensures a lower ambient temperature during the extraction process. The enhancement of molecular diffusivity in the subcritical state can enhance mass transfer, improve the permeability and solubility of substances during the extraction process, and prevent the decomposition of thermally unstable components at lower temperatures. Compared with SFE, it also has the advantages of low cost and large-scale production capability (Qi, 2012). Recently, an increasing number of studies have focused on the extraction of SCFEs. For example, Li and Li (2019) used a subcritical butane solvent to extract dried flowers of bitter roses, and the extraction rate attained 2.56% following optimization. Wang et al. (2016) compared the extraction of lavender extract using SCFE with that of traditional extraction methods. SCFE significantly increased the yield of lavender extract, and the lavender absolute was obtained by optimizing the ethanol extraction process (controlling the ratio of temperature-to-ethanol solution). The problems of low linalool content and a high content of harmful camphor in lavender absolute was solved. Zhang and Wu. (2016) compared the effects of SCFE, SFE, and ethyl acetate solvent extraction on the extraction of dandelion absolute. The results of GC-MS analysis showed that SCFE had higher extraction efficiency for aldehydes and acids, SFE had higher extraction efficiency for ketones and alcohols, and ethyl acetate extraction had higher extraction efficiency for esters; therefore, the extraction method should be selected according to the key components in the extract.

In industrial production, certain difficult problems with SCFE remain. Compared with maceration, SCFE requires more equipment input and a larger occupation area and its production capacity is lower. Thus, there is a need for further research and improvements.

### 3.7 Pressurized liquid extraction

**FIGURE 6:**
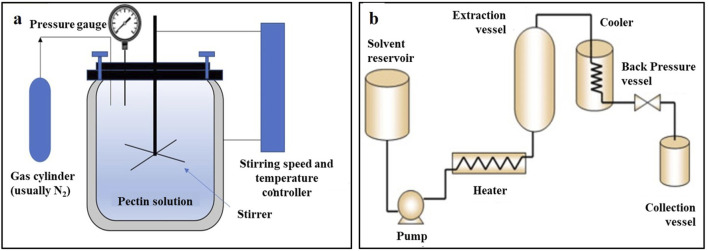
Simplified diagrams of **(A)** subcritical fluid and **(B)** pressurized liquid extractions (Basak and Annapure, 2022).

Pressurized liquid extraction (PLE) is also known as pressurized fluid extraction. A schematic diagram of the extraction equipment is shown in Figure 6B. Under a high pressure, the organic solvent remains near the supercritical region, thus maintaining a liquid state. Simultaneously, the surface tension and viscosity of the solvent decrease, and the permeability of the extraction matrix increases, thus increasing the efficiencies of mass transfer and diffusion. The extraction time and solvent consumption are also decreased, and the destruction of plant cell vacuoles and cell membranes under high pressure facilitates the target compounds easier to extract (Cascaes Teles et al., 2021). At high temperatures and pressures, the dielectric constant of water changes. Moreover, the intensification of the movement of water molecules results in a decrease in intermolecular hydrogen bonds, which leads to lower polarity, rendering it possible to use water as an extraction solvent and greatly reducing the cost of the solvent. This principle has been applied to pressurized hot solvent extraction and subcritical water extraction. Su and Zhang (2020) used PLE to extract Maojian and Qianliang tea extracts. Compared with unpressurized maceration, the extraction rate of Maojian tea extract *via* PLE was 3.12% higher, and the yield of Qianliang tea extract increased by 1.77%, which demonstrated that PLE can promote the extraction of plant components.

However, the decomposition of thermally unstable compounds at high temperatures is unavoidable, thus limiting the application of PLE. Several studies have attempted to avoid this weakness of PLE. Considering the thermally unstable compound paclitaxel in yew extract, Zhao and Wang (2020) soaked the mixture in an ethanol aqueous solution and improved the extraction rate. Subsequently, they used the low-boiling point solvent ethyl acetate for PLE to avoid the decomposition of paclitaxel at higher temperatures. This principle is consistent with that of SCFE, and it is often said that SCFE is an optimized method of PLE.

Similar to that for SFE, PLE involves high equipment and operating costs. Under heating and pressure conditions, the mass transfer effect between the solvent and matrix is better, but it will cause the decomposition of thermally unstable compounds. Although this problem can be avoided under low-temperature conditions, the compound extraction rate will be lower.

**FIGURE 7:**
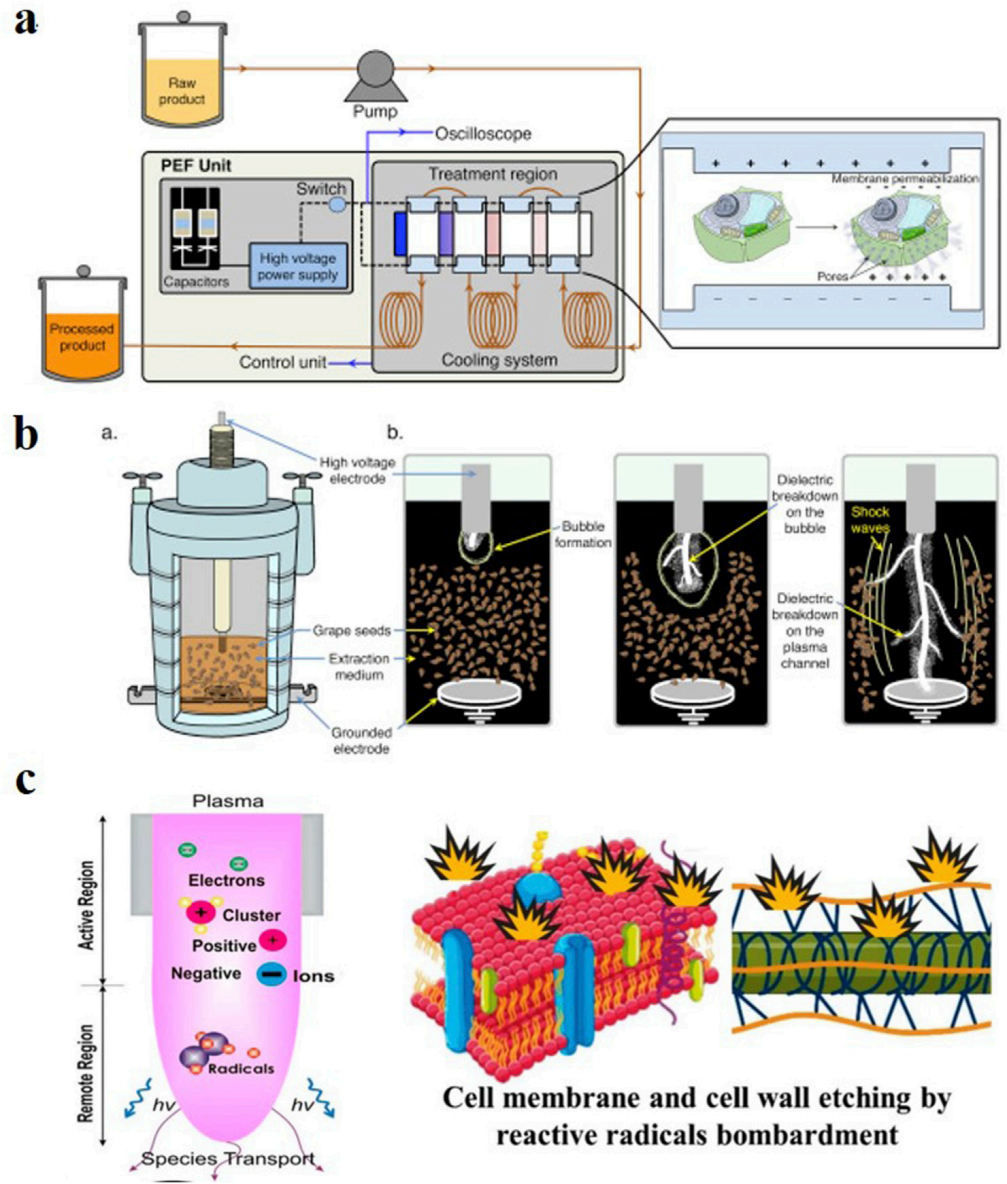
Schematic diagrams of a **(A)** pulsed electric field, **(B)** high voltage electric discharge, and **(C)** cold plasma-assisted extraction (Heydari et al., 2023; Barba et al., 2016).

As shown in Figure 7A, a PEF uses high-voltage electric fields to achieve the electroporation of cell membranes, thereby increasing solvent permeability and enhancing material transport. It applies short electrical pulses to plant cells over a short period. The cell membrane is exposed to an electric field and generates an electric field potential that causes electrostatic charge separation, resulting in the electroporation of the cell membrane and the generation of pores that promote the diffusion of intracellular substances (Käferböck et al., 2020; Naliyadhara et al., 2022). Luo and Wang (2022) used a PEF to extract *Pyracantha fortuneana* extract. Compared to the reflux extraction method, the PEF extraction effect (comprehensive calculation based on total flavonoids, polyphenols, red pigments, and extract yield) increased by 29%, confirming the high extraction rate of a PEF. Compared with MAE, a PEF does not require heating to destroy plant cell structures and avoids the generation of reactive oxygen species in UAE, thereby improving the extraction efficiency while protecting unstable components. In addition, using a PEF has the advantages of short processing time and low energy consumption (Bozinou et al., 2019). However, in some studies, the extraction rate of certain matrices decreased under higher electric field strengths, but the cause of this phenomenon has not yet been determined (Naliyadhara et al., 2022).

As shown in Figure 7B, HVED-assisted extraction is a new technology that uses the high-energy discharge of electrodes in aqueous media to generate local hotspots and plasma. This generates shock waves and a high turbulence to destroy plant cell walls, which enhances mass transfer and improves the extraction rate. Compared with PEF-assisted extraction, HVED-assisted extraction often has a better extraction efficiency in the extraction of algal components (Zhang et al., 2021), primarily because of the higher cell wall disintegration of HVED, whereas the opposite is true in fruit component extraction (Lončarić et al., 2020). This suggests that we need to consider the cellular structure of the extraction matrix when selecting an extraction method. However, there is currently no relevant research on the use of HVED for the extraction of plant extracts.

As shown in Figure 7C, CP-assisted extraction technology is an emerging method that uses CP to treat the matrix and improve the extraction rate of the target compounds (Yan and Li, 2023). Although relevant literature discusses its principles, they remain unclear. It is believed that the high-speed movement of particles in plasma is harmful to plants because it breaks down the cell wall (Bao et al., 2020) to enhance mass transfer and extraction. However, Ke et al. (2022) reported that CP treatment produces active ions and free radicals in the solvent, which react with certain components in the plant, resulting in a reduction in the extraction rate.

### 3.8 Electric field-assisted extraction

Electrically assisted extraction technology encompasses the application of electric or magnetic field technologies for plant extraction. It includes a pulsed electric field (PEF), high-voltage electric discharge (HVED), and cold plasma (CP).

Owing to its advantages of mild reaction conditions, environmental friendliness, simple operation, and economic benefits, CP-assisted extraction has been increasingly used in recent plant extraction research. For example, Bao et al. (2020) used CP to enhance the extraction of phenolic compounds from grape pomace. Xu and Wang (2023) used CP to pretreat ginger powder to improve the extraction rate of total flavonoids in the ginger powder extract. They also conducted scanning electron microscopy and infrared spectroscopy characterizations of the treated ginger powder and found that the cell wall surface of the treated ginger powder was rougher and had a reduced lignin content, which further promoted solvent penetration.

### 3.9 Other green extraction techniques

Currently, other studies have reported on new green technologies, but few related studies and applications, such as infrared-assisted extraction (IAE), flash extraction (FE), steam explosion extraction (SEE), and semi-bionic extraction (SBE), have been reported.

In IAE, after the substance to be extracted absorbs infrared light waves, its internal photon energy is converted into molecular vibration or rotational energy, causing the internal temperature of the substance to increase, thereby increasing the solubility of the target extract (Xiang et al., 2022). Compared with traditional extraction methods, IAE has a higher extraction rate and efficiency and lower investment cost and energy consumption than other green technologies. However, it has no advantage in extracting thermally unstable compounds and can easily trigger various chemical reactions.

FE is a method that uses high-speed rotational shear force and super-moving molecular percolation to quickly shear plant raw materials into particles, thereby greatly increasing the contact area between raw materials and solvents and improving the extraction efficiency (Xiafei et al., 2021). This method can be used for extraction under normal temperature conditions and is friendly for thermally unstable compounds; however, it is difficult to achieve large-scale industrial production.

SEE uses steam to heat natural products to 180°C–235°C, followed by maintaining the pressure for several seconds to minutes, and then instantly releases the steam. When the pressure is instantly reduced, secondary steam is generated to rapidly expand. This causes the plant tissues and cells to be destroyed by mechanical forces, thus increasing the extraction rate (Duque et al., 2016). Compared to FE, the SEE equipment cost and energy consumption are lower, which is conducive to large-scale industrial production. However, SEE is not conducive to the extraction of thermally unstable compounds and volatile components.

SBE is a new extraction technology that combines drug and molecular medicine research by simulating the transport process of natural products in the gastrointestinal tract and successively extracting them with aqueous solvents at a specific pH. It is often used for the extraction of traditional Chinese medicines. SBE does not require the use of organic solvents, does not produce solvent residues, has a low extraction temperature, and is compatible with thermally unstable components. However, the high cost and low extraction efficiency of the characteristic solvents render it difficult for SBE to become an industrially applicable technology, and its use in the extraction of certain precious traditional Chinese medicines is limited (Wang et al., 2016).

In summary, although various green extraction technologies have their own advantages compared with traditional extraction methods, they generally have the limitations of high operating costs and difficulty in achieving large-scale production. Moreover, they are only suitable for extracting components with high commercial value. Further reducing the operating costs of new technologies and enabling their faster application in large-scale production are important goals for the current development of green extraction technology. To facilitate the analysis and comparison of various methods, this review compares and discusses the advantages and disadvantages of each method, as shown in Table 2.

**TABLE 2:** Comparison of extraction techniques.

Extraction technology	Advantages	Disadvantages	Remarks
Maceration	simple operation, low energy consumption, low processing temperature, wide application range	time-consuming, low extraction rate, use of toxic organic solvents	The solvent can be selected based on the target components
Percolation	low process temperature, high extraction rate	time-consuming, high solvent consumption	Primarily used for the extraction of valuable, toxic compounds and high-concentration preparations
Reflux extraction	high extraction efficiency, low solvent consumption, avoids the diffusion of toxic components	not suitable for heat-labile ingredient extraction	Often used to extract volatile components from natural plants, and the extraction rate of non-volatile components is low
Soxhlet extraction	high extraction rate, high extraction efficiency, low solvent consumption	not suitable for heat-labile ingredient extraction	The Soxhlet extraction device makes the extraction operation simpler, and the internal parameters of the extraction stage are controllable
MAE	high extraction efficiency, low solvent loss, high degree of automation, uniform heating, green and environmentally friendly	poor extraction rate of non-polar or volatile compounds, not suitable for the extraction of thermally unstable components	Improvement measures in recent research: using nitrogen pressurization to prevent the oxidation of secondary metabolites, extracting solvents using vacuum to promote mass transfer, and reducing thermal decomposition and oxidation reactions
UAE	simple operation, low solvent consumption, high extraction efficiency, low processing temperature, green and environmentally friendly	reactive oxygen species generated by cavitation can cause the oxidation or degradation of certain compounds	The cavitation effect of ultrasonic waves is affected by physical properties such as solvent viscosity and surface tension
EAE	high extraction rate, low solvent consumption, mild extraction conditions, simple operation, green and environmentally friendly	high cost and time consuming	Biological enzymes are expensive and the environmental conditions for enzymatic activity are harsh; therefore, the extraction environment must be controlled
SFE	high extraction rate, no solvent residue, mild processing conditions, green and environmentally friendly	expensive equipment, high cost, and poor extraction effect on highly polar and high-molecular-weight substances	SFE does not have a good extraction effect on all components. Adding an entrainer can make up for this defect to a certain extent
SCFE	high extraction rate, wide application range, non-destructive extraction, mild processing conditions, green and environmentally friendly	high equipment investment, large floor area	SCFE makes it easier to achieve large-scale industrial production
PLE	high extraction rate, high extraction efficiency, wide application range, green and environmentally friendly	high equipment investment, difficulty in industrial application	High temperature and pressure will cause the decomposition of thermally unstable components, and the extraction rate will decrease at a low temperature and high pressure
PEF	high extraction efficiency, low energy consumption, mild processing conditions, green and environmentally friendly	high cost and small production scale	The extraction rate of certain raw materials will decrease under higher electric field strength, and the reason for this phenomenon has not yet been determined
HVED	high extraction efficiency, low solvent consumption, green and environmentally friendly	high cost, prone to electrochemical reactions, small production scale	HVED has different effects on different raw materials. For example, the damage effect on algae cells is better than that on fruit cells
CP	high extraction efficiency, low energy consumption, mild processing conditions, green and environmentally friendly	high cost, unclear extraction principle	CP will undergo complex reactions on the surface of plant cells, and the mechanism of action is still controversial
IAE	high extraction efficiency, low energy consumption, easy to achieve large-scale production, green and environmentally friendly	not advantageous for the extraction of thermally unstable compounds	IAE can easily cause the thermal decomposition of extracted ingredients and various other chemical reactions
FE	high extraction efficiency, low cost, mild processing conditions, green and environmentally friendly	not suitable for raw materials that are not easily crushed	—
SEE	high extraction efficiency, low cost, and conducive to large-scale industrial production	Not advantageous for the extraction of thermally unstable compounds	—
SBE	more biocompatible extracts, mild processing conditions, green and environmentally friendly	high cost, low extraction efficiency, difficulty in industrial applications	The components in the extract constructed by SBE are prone to react under acidic and alkaline environments

### 3.10 Collaborative extraction technology

The primary goal of any extraction technique is to maximize the recovery of target compounds from the sample matrix while maintaining the integrity of the molecules of interest and reducing the co-extraction of other impurities or undesirable compounds (Garcia-Vaquero et al., 2018). Therefore, selecting an extraction technique based on the target components is critical. However, for extracts and absolutes with complex components, no single extraction technology can effectively extract all the compounds from plants, and extraction technology is limited by various factors, such as the economic value of the compounds and environmental issues. Currently, an extraction technology that combines all the advantages of these technologies without limitations has yet to emerge. Collaborative extraction technologies can overcome the limitations of a single method and integrate the advantages of various extraction methods. This is a feasible solution for the high-quality extraction of plant extracts and absolutes.

He and Huang (2023) developed a method for obtaining tobacco extracts that combined CP and IL technologies. They used CP to, etch the cell surface to rupture the cell wall and then used an ILs to dissolve cellulose to further improve the extraction rate. The obtained extract was diluted and added to cigarettes. After comparing the smoke evaluation, the extract obtained using this method was significantly better than the reflux extraction method and separate extraction products of CP and IL in terms of style, taste, comfort, smoke characteristics, and aroma. Tian et al. (2023) combined the EAE and UAE methods to extract *Calophyllum inophyllum L*. and used it to flavor cigarettes. Following treatment with complex enzyme preparations (including cellulase, pectinase, and xylanase), part of the cell wall was enzymatically prepared. The enzymatic reaction produced new flavor substances during decomposition, and UAE further improved the extraction rate of various components. The crab-apple extract obtained using this method was flavored in cigarettes and received unanimous praise from 33 professional sensory evaluators. Zeng et al. (2015) explored the mechanisms of two green extraction technologies, ultrasonic-assisted extraction (UAE) and microwave-assisted extraction (MAE). They utilized the cavitation bubbles produced by the vibrations in UAE and the high energy from MAE to expedite the rupture of plant cells, leading to a faster release of beneficial compounds. This approach not only improved the yield but also shortened the extraction time and decreased the need for solvents. Wang et al. (2014) investigated the use of ionic liquids (IL) as replacements for traditional extraction solvents, combining UAE and MAE in their extraction process. Their findings indicated that although the content of certain compounds increased with higher IL ion concentrations, the overall extraction rate decreased when the concentration became too high. This demonstrates the selective nature of IL as an extraction solvent and highlights the fact that merely combining different extraction technologies or solvents does not guarantee enhanced extraction efficiency; each combination must be analyzed based on specific results. Similarly, Osorio-Tobon et al. (2014) conducted a study that combined pressurized liquid extraction (PLE) and supercritical fluid extraction (SFE) to extract turmeric. They observed that as pressure increased, the extraction efficiency for turmeric diminished. This may be attributed to a reduction in the active surface area of the raw material under excessive pressure, which decreases the contact area between the solvent and the target compounds. In contrast, studies involving the combination of SFE and UAE, as well as UAE and PLE, showed no decrease in extraction rates (Sumere et al., 2018; Santos et al., 2015). Therefore, it can be concluded that the integration of different extraction technologies can yield unexpected results; for example, the extraction rate may not increase as anticipated but may actually decrease. While some studies have provided possible explanations for these observations, a more authoritative and credible explanation is needed to clarify the specific causes.

## 4 Conclusion

This article reviews the traditional and advanced green extraction technologies of plant extracts and absolutes and conducts a comparative analysis. Because food safety is increasingly valued, the demand for natural plant extracts in the pharmaceutical, food, flavoring, and fragrance industries is increasing. Given the requirements of green chemistry and a high extraction rate, it is necessary to replace traditional extraction technologies with advanced green extraction technologies. However, the current green extraction technology remains limited, and problems such as high-cost investment and low production capacity render it difficult to achieve large-scale industrial production. Developing and improving green extraction technology and making it suitable for large-scale industrial production to meet the future demand for plant extracts is currently the primary goal. In addition, the selection of the extraction technology should be carefully considered because different extraction technologies have both advantages and disadvantages. Factors such as the extraction of raw materials, extraction technology characteristics, extraction rate, efficiency, cost, and key components of the extract should be comprehensively considered. This study provides suggestions on the selection of a green extraction method.

I. The key components of plant extracts and absolutes include the key aroma components of spices and the key active components of medicinal materials. If the key components are thermally unstable compounds, avoid using MAE, PLE, SEE, and IAE with high extraction temperatures and choose methods with mild industrial conditions, such as UAE, EAE, and SFE. If the key ingredients are prone to redox reactions, the use of UAE, which generates reactive oxygen species, HVED, and IAE, which can easily cause various chemical reactions, should be avoided.
II. From the perspective of the extraction rate and efficiency, green extraction methods generally have higher extraction rates than do traditional methods. However, the extraction efficiency varies. Considering MAE, UAE, PLE, and other methods as examples, the extraction efficiencies are high, whereas EAE and SBE have low extraction efficiencies. Methods with high extraction efficiencies are often associated with severe processing conditions. However, SFE and PEF can achieve high extraction efficiency under mild conditions and can be used as extraction methods for the large-scale production of high-value extracts when their extraction costs are high.
III. From the perspective of suitability for industrial production, the EAE, SCFE, IAE, and SEE methods can be used for large-scale extraction at the process level. However, at the cost and investment levels, the cost of EAE enzymes is high, and the investment in SCFE equipment is high, whereas IAE and SEE have relatively more advantages. In addition, constructing enzyme-producing microorganisms using molecular biology methods and performing microbial fermentation extraction are worthwhile solutions for reducing EAE costs and achieving large-scale industrial extraction. However, for certain high-value extracts, particularly the pharmaceutical extracts used to treat human diseases, the more expensive but more biocompatible EAE and SBE methods should be chosen.
